# Increased orexin A concentrations in cerebrospinal fluid of patients with behavioural variant frontotemporal dementia

**DOI:** 10.1007/s10072-021-05250-x

**Published:** 2021-04-27

**Authors:** Fausto Roveta, Andrea Marcinnò, Riccardo Cremascoli, Lorenzo Priano, Stefania Cattaldo, Elisa Rubino, Erica Gallo, Silvia Boschi, Alessandro Mauro, Innocenzo Rainero

**Affiliations:** 1 Department of Neurosciences “Rita Levi Montalcini”, University of Torino, Turin, Italy; 2 Department of Neurology and Neurorehabilitation, Istituto Auxologico Italiano, IRCCS, San Giuseppe Hospital, Piancavallo, Italy; 3 Department of Brain and Behavioural Sciences, University of Pavia, Pavia, Italy; 4 Laboratory of Clinical Neurobiology, Istituto Auxologico Italiano, IRCCS, San Giuseppe Hospital, Piancavallo, Italy; 5 Department of Neuroscience and Mental Health, AOU Città della Salute e della Scienza di Torino, Turin, Italy

**Keywords:** Orexin A, Hypocretin-1, Frontotemporal dementia, Cerebrospinal fluid

## Abstract

Orexins are hypothalamic neuropeptides that regulate several physiological functions, such as appetite, arousal, cognition, stress, sleep and metabolism. Emerging pieces of evidence suggest an orexinergic dysfunction in several neuropsychiatric disorders, including depression, anxiety and addiction. A syndromic overlap between behavioural variant frontotemporal dementia (bvFTD) and several psychiatric disorders was recently demonstrated. Therefore, we analysed cerebrospinal fluid (CSF) orexin A concentrations of 40 bvFTD and 32 non-demented patients, correlating neuropeptide concentrations with several clinical characteristics. A significant increase of orexin A concentrations was found in bvFTD patients when compared to controls (*p*<0.001). CSF orexin A concentration showed a correlation with Mini-Mental State Examination scores, drug assumption, history of compulsive behaviour and extrapyramidal signs. Moreover, we found a relationship between CSF markers of neurodegeneration, total tau and Aβ_1–42_ and CSF orexin A concentrations. Our study provides evidence of an orexinergic dysfunction in bvFTD, correlating with several clinical symptoms. Further larger studies are needed to confirm our data.

**Supplementary Information:**

The online version contains supplementary material available at 10.1007/s10072-021-05250-x.

## Introduction

Behavioural variant frontotemporal dementia (bvFTD) is the most common clinical variant within the spectrum of frontotemporal lobar degeneration syndromes and is characterized by personality changes, apathy, decline in socially appropriate behaviour, and empathy. These symptoms reflect progressive deregulation of the neural circuits involved in social cognition, emotion regulation, motivation and decision making [1].

Orexins (orexin A and orexin B) are two neuropeptides synthesized by hypothalamic neurons with widespread projections throughout the central nervous system. Orexins regulate several physiological functions, like appetite, arousal, cognition, stress, sleep and metabolism. Several studies suggested a role for orexins in psychiatric disorders, including addiction, depression and anxiety [2]. Moreover, the modulation of the orexinergic system may also have a role in sleep disorders as well as rehabilitation of traumatic brain injury [3]. Recently, some studies evaluated cerebrospinal fluid (CSF) orexin levels in Alzheimer’s disease (AD), finding a high level of orexin A that correlates with AD progression, sleep fragmentation and neuropsychiatric symptoms [4].

Studies about orexins in bvFTD are few and not conclusive. A study with magnetic resonance imaging (MRI) of early-FTD patients and post-mortem analyses of bvFTD cases showed significant atrophy of the posterior hypothalamus with orexinergic neuron sparing [5]. In a small group of bvFTD patients, CSF orexin A concentrations were not significantly different from controls, but a negative correlation between neuropeptide concentrations and daytime somnolence was found [6]. Finally, a recent study showed that CSF pro-orexin levels are increased in FTD as well as in AD patients compared to controls [7].

Orexinergic system dysregulation could represent a neurobiological basis of certain clinical and behavioural features of patients with frontotemporal dementia. Thence, considering the above-mentioned observations, the purpose of this study was to better investigate the role of orexinergic system in bvFTD through the measurement of CSF orexin A concentrations and the evaluation of possible correlations with clinical symptoms.

## Materials and methods

### Patients

A group of 40 bvFTD consecutive patients (18 males and 22 females, mean age: 68.27 ± SD: 8.61 years), evaluated and treated at the Department of Neuroscience of University Hospital “Città della Salute e della Scienza” of Torino and at the Department of Neurology and Neurorehabilitation of “S. Giuseppe” Hospital, Piancavallo, Italy, were recruited for the study. Diagnosis of probable bvFTD was made according to Rascovsky et al. criteria [8]. For each patient, neuroimaging findings with magnetic resonance imaging (MRI) and positron emission tomography with ^18^fluorodeoxyglucose (^18^FDG-PET) were available. All patients underwent lumbar puncture with measurement of CSF levels of orexin A, Aβ_1–42_, *p*-tau and *t*-tau. Genetic variants in the MAPT, PGRN and C9orf72 genes were excluded. Cognitive functions were assessed by a standard neuropsychological evaluation, including Mini-Mental State Examination (MMSE). Detailed clinical history was recorded particularly focusing on cognitive and behavioural characteristics, drugs assumption and cardiovascular risk factors. A group of 32 cognitively healthy subjects (14 males and 18 females, mean age ± SD: 63.30 ± 14.21 years) examined for neurological conditions other than dementia (e.g. suspected polyneuropathy or multiple sclerosis) was used as controls. A complete description of the above-mentioned clinical procedures is available in the Supplementary materials.

### CSF sample collection and biochemical analysis

All CSF samples were obtained by lumbar puncture, using an atraumatic needle, performed early in the morning (between 9.00 and 11.00 a.m.) after overnight fasting [9].

The CSF *t*-Tau, *p*-Tau_181_ and Aβ_1-42_ levels were measured separately, in duplicate, using commercially available sandwich enzyme-linked immunosorbent assays (ELISA) kits (Innnotest; Innogenetics/Fujirebio, Ghent, Belgium) according to the manufacturer’s instructions. Intra-assay and inter-assay coefficients of variation were 3.2% and 11.5% for *t*-tau, 1.7% and 11.4% for *p*-tau, 4.6% and 7.8% for Aβ_1–42_. The CSF Orexin levels were detected with the commercially available EIA kit (Phoenix Pharmaceuticals, Burlingame, CA, USA), based on the principle of competitive enzyme immunoassay. The intra-assay and inter-assay coefficients of variation were 7.5% and 5.8%, respectively. All samples were assayed in duplicate.

### Statistical analysis

Continuous variables were described as mean and standard deviation or medians and range, and categorical ones as count and percentage. D’Agostino-Pearson’s test was used to assess the normality of included variables. Differences of continuous data between groups were studied by the unpaired *t*-test or its non-parametric variant (Mann-Whitney test), while differences of categorical variables by the Chi-square test with Yate’s correction. Correlation between continuous variables was analysed through Pearson or Spearman (in case of non-normal distribution) correlation. Multivariate generalized linear models (GLMs) were performed to estimate the quantitative relationship between CSF orexin A concentration and other variables considered as independent predictors. All analyses were run with R software (www.r-project.org). The level of statistical significance was defined at *p* < 0.05.

## Results

**Fig. 1:**
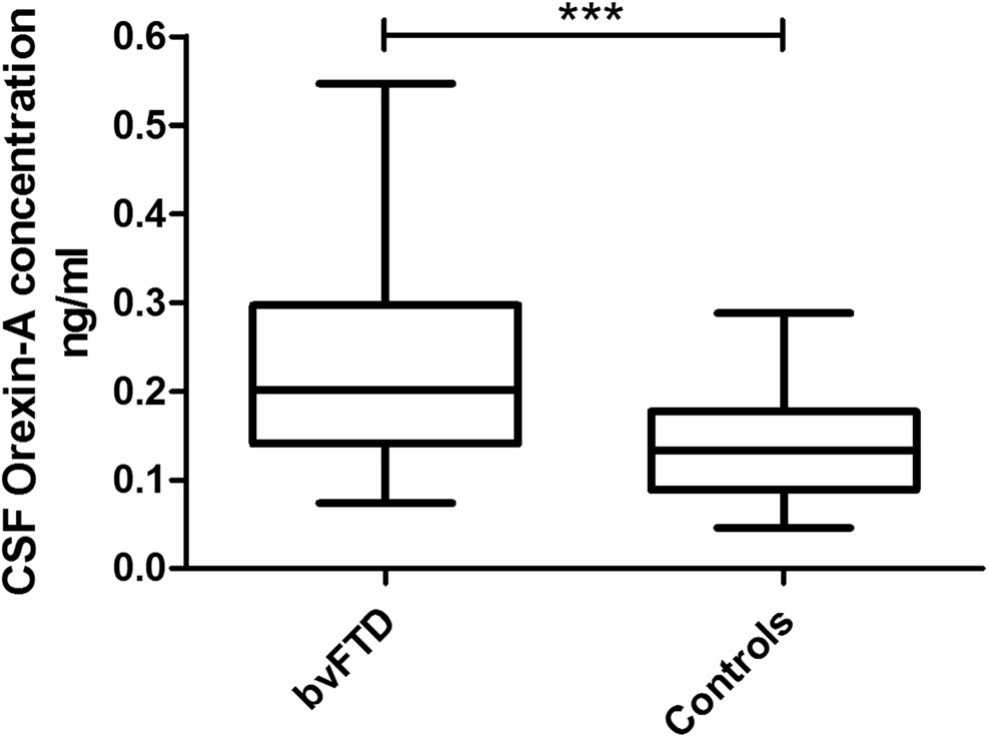
CSF levels of orexin A detected in bvFTD and control groups. Box plot representing median, interquartile range and range (maximum-minimum) of CSF orexin A levels in bvFTD and control groups. ***p < 0.001

We found increased CSF orexin A concentrations in bvFTD patients (mean: 0.226 ng/mL ± SD: 0.103) compared to controls (mean: 0.139 ng/mL ± SD: 0.058), and this resulted statistically significant (*p* < 0.001) (Fig. 1), also when adjusted for age and sex (*p* < 0.001).

In addition, several clinical and demographic variables emerged as significantly linked to CSF orexin A concentrations in the bvFTD group (Table 1). We found a significant inverse correlation with MMSE, in both univariate and multivariate analyses (*p* < 0.001), while age at lumbar puncture and disease duration did not correlate with orexin A concentrations. In multivariate analysis, the presence of repetitive/compulsive behaviours as well as extrapyramidal signs was associated with increased CSF orexin A concentrations. Finally, neurodegenerative biomarkers of dementia, CSF *t*-Tau and Aβ_1–42_ concentrations co-variated with orexin A concentrations, though in the reciprocally opposite sense.

**Table 1:** Demographic characteristics and CSF biomarkers of bvFTD and control groups (count and percentage or mean ± SD) and clinical variables significantly linked to orexin A concentrations in bvFTD patients

**Demographic and clinical characteristics**	**bvFTD**	**Controls**	***p*** **value**
Gender M/F (%)	18/22 (45/55)	14/18 (43.75/56.25)	0.89
Age (years)	68.27 ± 8.61	62.30 ± 14.21	0.06
Disease duration (years)	3.52 ± 1.95	-	-
MMSE	23.55 ± 3.45	-	-
**AD—neurodegeneration CSF biomarkers**	**bvFTD**	**Controls**	***p*** **value**^**a**^
*t*-tau	182.39 ± 182.68	106.85 ± 77.41	0.06
*p*-tau	44.78 ± 24.65	26.18 ± 14.28	0.001
Aβ_1–42_	787.43 ± 269.63	949.28 ± 315.07	0.11
**Multivariate analysis—predictors of orexin A concentrations in bvFTD**	**Std. coefficient for orexin A**		***p*** **value**
MMSE	−0.6751		< 0.001
Repetitive/compulsive behaviour	0.3445		< 0.05
Extrapyramidal signs	−0.3583		< 0.01
SSRI/SNRI assumption	−0.2744		< 0.05
*t*-tau	−0.4156		< 0.01
Aβ_1–42_	0.3101		< 0.05

^a^After adjustment for age and sex

## Discussion

In this study, we found that CSF orexin A concentrations are significantly increased in bvFTD patients in comparison to controls. In addition, we found that neuropeptide concentration correlated with several clinical characteristics of the disease, like repetitive/compulsive behaviour and extrapyramidal signs. Finally, a correlation between markers of neurodegeneration and CSF orexin A concentrations was found. Our data are in accordance with a previous study [7] showing increased CSF pro-orexin concentrations in a group of 32 FTD patients. On the contrary, Liguori and colleagues [6] found no difference in orexins concentrations comparing a small group of FTD patients (*n* = 11) with controls. However, this study was conducted in patients with a diagnosis of FTD syndrome, while our study included only bvFTD patients according to Rascovsky criteria.

Increased orexin A concentrations in bvFTD patients observed in our study may have several neurobiological explanations as well as important clinical implications. Interconnections between cortical areas and orexinergic neurons are increasingly under investigation, especially with concern to feeding and addiction disorders [10]. The prefrontal cortex emerges as a possible inhibitory interactor of hypothalamic orexinergic activity, and prefrontal disruption could mediate orexinergic tone deregulation, explaining higher orexin A concentrations in bvFTD patients.

We found an intriguing correlation between repetitive/compulsive behaviour and CSF orexin A concentrations. Several animal-model studies showed suppression of compulsive behaviours linked to abuse substances by orexin receptor antagonism [11]. Although pieces of evidence derived by human observational studies are fewer in number [12], we suggest a role of orexinergic system in enhancing motivation-linked compulsive behaviours, as they could be found in bvFTD patients. In addition, the presence of extrapyramidal signs showed to be an independent predictor of lower orexin A concentrations. This finding is consistent with previous pieces of evidence: in PD animal models orexin A demonstrated neuroprotective effects on dopaminergic neurons in substantia nigra [13], while orexinergic and dopaminergic neuronal loss was correlated with PD duration and progression in human autoptic cases [14]. Moreover, repeated CSF orexin measurements in PD patients revealed a decrease of levels over years and objective sleepiness correlated with decrease of CSF orexin levels [15], so suggesting that both orexin and dopamine deficiencies, and dopaminergic stimulation, may affect sleep and wakefulness in this neurodegenerative disorder [16, 17].

Finally, we found that CSF orexin A concentrations correlated with markers of neurodegeneration like Aβ_1–42_ and total tau. Preclinical and clinical studies suggested a role for Aβ_1–42_ in regulating amyloid clearance and, consequently, Alzheimer’s disease progression [18]. Furthermore, the negative correlation found with *t*-tau reinforces a role for this neuropeptide in neurodegenerative processes. Further studies are warranted in order to evaluate the potential role of orexin A as a biomarker of bvFTD.

Our study certainly has some limitations. For example, the low number of enrolled patients must be acknowledged. However, even if preliminary, correlations between orexin A concentrations and cognitive status, as well as motor and psychiatric symptoms, deserve additional investigations. To our knowledge, this is the largest clinical series demonstrating an increase of orexin A concentrations in cerebrospinal fluid of bvFTD patients. Moreover, it was possible to report several associations with the clinical characteristics of the disease. Better cognitive performance appeared to be a predictor of lower orexin A, thus reinforcing the hypothesis of a deregulation of this neuropeptide in the disease. Regarding specific neuropsychiatric manifestations, we found significant orexin A variations when independently assessing for a history of compulsive behaviours or extrapyramidal signs. In both cases, a possible explanation is based on orexinergic physiological functions and interconnections with other neurotransmitter systems. Finally, also neurodegenerative process, as delineated by CSF markers, emerged as an independent predictor of orexin A variability in this study, suggesting a complex role of neuronal loss in deregulating the cognitive-behavioural functions related to orexinergic system.

## Supplementary Information

ESM 1 (DOCX 25 kb)

## Abbreviations

bvFTD: Behavioural variant frontotemporal dementia
AD: Alzheimer disease
PD: Parkinson’s disease
