# Supplementary material for: Increased orexin A concentrations in cerebrospinal fluid of patients with behavioural variant frontotemporal dementia
Source: Neurol Sci. 2021 Apr 27;43(1):313–7. doi: 10.1007/s10072-021-05250-x (PMC8724071; doi:10.1007/s10072-021-05250-x)
Supplement: Supplementary file 1 — (DOCX 25 kb) [file 10072_2021_5250_MOESM1_ESM.docx]

*Supplementary materials*

**Increased Orexin-A Concentrations in Cerebrospinal Fluid of Patients with Behavioural Variant Frontotemporal Dementia**

**APPENDIX A**

**A.1. CSF Collection and analysis**

All CSF samples were obtained after overnight fasting early in the morning (between 9.00 and 11.00 a.m.) by lumbar puncture using an atraumatic needle ([Doherty](https://www-ncbi-nlm-nih-gov.bibliopass.unito.it/pubmed/?term=Doherty%20CM%5BAuthor%5D&cauthor=true&cauthor_uid=25075138)et al. 2014).

CSF samples were collected in polypropylene tubes using standard sterile techniques. CSF samples were centrifuged to eliminate cells and cellular debris, and immediately frozen at -80°C to measure orexin-A, t-tau, p-tau Aβ_1-42_levels.

CSF orexin-A concentrations were detected according to previously published standard procedures with commercially available ELISA kit (Orexin A/Hypocretin-1 EIA kit; Phoenix Pharmaceuticals, Burlingame, CA), based on the principle of competitive enzyme immunoassay. The unknown sample concentrations were calculated on the corresponding standard sigmoid curve equation; the pathologic cutoff level was set at ≤ 50 pg/mL (Liguori et al., 2014, Liguori et al., 2019).

CSF Aβ_1-42_, t-tau and p-tau levels were determined using commercially available sandwich enzyme-linked immunosorbent assays (ELISA; Innotest b-Amyloid 1-42, Innotest h-T-tau, Innotest Phospho-T-tau 181; Fujirebio Ghent, Belgium).

Normal values of biomarkers were considered as follows: Aβ_1-42_>500 pg/mL; tau<300 pg/mL; p-tau< 61pg/ mL (Sjogren M et al., 2001; Vanderstichele H. et al., 2006; Sauvée et al., 2014).

**APPENDIX B**

The following codes define different significance levels: “***” for p < 0.001; “**” for p < 0.01; ”*” for p < 0.05.

**Table B.1. Clinico-demographic characteristics.**

| **Demographic characteristics** | **bvFTD** | **Controls** | | **p value** | |
| --- | --- | --- | --- | --- | --- |
| Gender - M/F (%) | 18/22 (45.0/55.0) | 14/18 (43.75/56.25) | | 0.89^a^ | |
| Gender (M/F) - Or-A mean (ng/mL) ± SD | 0.232 ± 0.124/  0.221 ± 0.084 | 0.145 ± 0.064/  0.134 ± 0.055 | | 0.74 (bvFTD)^b^  0.59 (OTD)^b^ | |
| Age at LP (years) | 68.27 ± 8.61 | 62.30 ± 14.21 (n=28) | | 0.53 (bvFTD)^c^  0.44 (OTD)^c^ | |
| Disease duration (years) | 3.52 ± 1.95 | _ | | 0.54^c^ | |
| MMSE | 23.55 ± 3.45 | _ | | 0.01* ^c^ | |
| **Clinical characteristics (bvFTD)** | **Count (%)** | **Or-A mean ± SD if condition was** | | | |
|  |  | **present** | **absent** | | **P value^b^** |
| Neurodeg. familiarity | 24/39 (61.54) | 0.201 ± 0.080 | 0.268 ± 0.126 | | 0.08 |
| Hypertension | 17/39 (43.59) | 0.230 ± 0.090 | 0.224 ± 0.116 | | 0.51 |
| Type 2 diabetes | 3/39 (7.69) | 0.200 ± 0.067 | 0.229 ± 0.107 | | 0.77 |
| Smoke | 15/39 (38.46) | 0.225 ± 0.086 | 0.228 ± 0.116 | | 0.89 |
| Chronic CVD | 19/39 (48.72) | 0.201 ± 0.081 | 0.252 ± 0.119 | | 0.27 |
| SSRI/SNRI | 24/40 (60.00) | 0.209 ± 0.079 | 0.251 ± 0.130 | | 0.26 |
| Antipsychotics | 11/40 (27.50) | 0.230 ± 0.114 | 0.224 ± 0.100 | | 0.84 |
| AChE-I | 4/40 (10.00) | 0.311 ± 0.173 | 0.216 ± 0.091 | | 0.23 |
| Insomnia | 13/40 (32.50) | 0.223 ± 0.099 | 0.221 ± 0.115 | | 0.94 |
| Daytime sleep | 6/40 (15.00) | 0.244 ± 0.120 | 0.223 ± 0.101 | | 0.70 |
| Depression | 26/40 (65.00) | 0.220 ± 0.106 | 0.237 ± 0.010 | | 0.49 |
| Apathy | 31/40 (77.50) | 0.233 ± 0.110 | 0.201 ± 0.076 | | 0.42 |
| Disinhibition | 20/40 (50.00) | 0.201 ± 0.082 | 0.251 ± 0.117 | | 0.12 |
| Rep/comp behaviour | 28/40 (70.00) | 0.222 ± 0.090 | 0.234 ± 0.133 | | 0.99 |
| Hyperphagia | 7/40 (17.50) | 0.220 ± 0.106 | 0.227 ± 0.104 | | 0.78 |
| Extrapiramidal disturb | 16/40 (40.00) | 0.224 ± 0.123 | 0.227 ± 0.090 | | 0.68 |
|  |  |  |  |  |  |

CVD: cerebrovascular disease; CNS drugs: having their primary mechanism of action in central nervous system; SSRI/SNRI: selective serotonin/norepinephrine reuptake inhibitors; AChE-I: cholinesterase inhibitors; LP: lumbar puncture; MMSE: mini-mental status examination.

^a^ Chi-square test with Yate’s correction used to compare sex distribution in bvFTD and ODT groups.

^b^ Unpaired t (with Welch’s correction in case of unequal variances) or Mann-Whitney test (in case of non normal distribution) were performed to compare mean CSF Orexin-A concentrations in case of presence or absence of the variable indicated in first column.

^c^  Pearson or Spearman (in case of non normal distribution) correlation were performed to study the correlation between CSF Orexin-A concentration and each continuous variable indicated in first column.

**Table B.2. Best fitting GL model applied to Or-A in bvFTD patients^e^**

|  | Estimated coefficient^f^  for Or-A (ng/mL) | Std. Error | t value | p value |
| --- | --- | --- | --- | --- |
| MMSE | -2.082e-02 | 4.164e-03 | -4.999 | 2.78e-05*** |
| Neurodeg. familiarity | -6.012e-02 | 2.562e-02 | -2.346 | 0.02627* |
| Type 2 diabetes | 6.805e-02 | 4.667e-02 | 1.458 | 0.15595 |
| CNS drugs | 6.138e-02 | 3.282e-02 | 1.870 | 0.07192 |
| SSRI/SNRI use | -5.728e-02 | 2.491e-02 | -2.299 | 0.02915* |
| AChE-I use | 1.328e-01 | 3.936e-02 | 3.374 | 0.00218** |
| Rep/comp behaviour | 7.663e-02 | 2.800e-02 | 2.737 | 0.01064* |
| Extrapiramidal disturb | -7.480e-02 | 2.658e-02 | -2.814 | 0.00886** |
| t-tau | -2.343e-04 | 6.720e-05 | -3.486 | 0.00164** |
| Aβ_1-42_ | 1.199e-04 | 5.238e-05 | 2.288 | 0.02986* |

MMSE: mini-mental status examination; Neurodeg. familiarity: familiar history of neurodegenerative disorders; CNS drugs: having their primary mechanism of action in central nervous system; SSRI/SNRI: selective serotonin/norepinephrine reuptake inhibitors; AChE-I: cholinesterase inhibitors; rep/comp behaviour: history of behaviour disorder including repetitive or compulsive manifestations

**References**

- [Doherty CM](https://www-ncbi-nlm-nih-gov.bibliopass.unito.it/pubmed/?term=Doherty%20CM%5BAuthor%5D&cauthor=true&cauthor_uid=25075138),[Forbes RB](https://www-ncbi-nlm-nih-gov.bibliopass.unito.it/pubmed/?term=Forbes%20RB%5BAuthor%5D&cauthor=true&cauthor_uid=25075138).Diagnostic Lumbar Puncture. [Ulster Med J](https://www-ncbi-nlm-nih-gov.bibliopass.unito.it/pubmed/25075138) 2014;83(2):93-102.
- Liguori C, Romigi A, Mercuri NB, Nuccetelli M, Izzi F, Albanese M, Sancesario G, Martorana A, Sancesario GM, Bernardini S, Marciani MG, Placidi. Cerebrospinal-fluid orexin levels and daytime somnolence in frontotemporal dementia. J Neurol 2014; 261:1832-1836.
- Liguori C, Moresco M, Izzi F, Mercuri NB, Plazzi G, Placidi F. New revolution in the assessment of cerebrospinal fluid orexin-A: Enzyme-linked immunosorbent assay! Psychiatry Clin Neurosci 2019; 73: 194-195.
- Sjögren M, Vanderstichele H, Agren H, et al. Tau and Abeta42 in cerebrospinal fluid from healthy adults 21-93 years of age: establishment of reference values. Clin Chem. 2001;47(10):1776-1781.
- Vanderstichele H, De Vreese K, Blennow K, et al. Analytical performance and clinical utility of the INNOTEST PHOSPHO-TAU181P assay for discrimination between Alzheimer's disease and dementia with Lewy bodies. Clin Chem Lab Med. 2006;44(12):1472-1480.
- Sauvée, M., DidierLaurent, G., Latarche, C., Escanyé, M. C., Olivier, J. L., & Malaplate-Armand, C. (2014). Additional use of Aβ 1-42/Aβ 1-40 ratio with cerebrospinal fluid biomarkers P-tau and Aβ1-42 increases the level of evidence of Alzheimer's disease pathophysiological process in routine practice. Journal of Alzheimer's disease : JAD, 41(2), 377–386.
